# Dr. William R. Campbell

**Published:** 1907-05

**Authors:** 


### OBITUARY.

DR. WILLIAM R. CAMPBELL, of Niagara Falls, died at his home in that city April 9, 1907, of cancer of the liver, aged 53 years. He was a native of New York State, his family residing at Trumansburg for many years. He graduated in medicine at the University of Buffalo in 1880, and immediately thereafter began practice at Niagara Falls, being associated for many years with the late Dr. Gardner C. Clarke. He was one of the most prominent physicians in the city of his residence and was likewise interested in many business enterprises.

Dr. Campbell was the reigning president at the time of his death of the Alumni Association of the medical department of the University of Buffalo; he was also a member of the various local medical societies and was a medical officer in the National Guard holding the rank of captain.

He is survived by his wife, four sisters, Mrs. I. M. Peck and Mrs. Warren Peirson of Trumansburg, N. Y., Mrs. Frederick Trumbell of Ithaca and Miss Maud Campbell of this city, and three brothers, Daniel W. Campbell of this city, Dr. Alexander J. Campbell of Syracuse and James Campbell of Trumansburg, N. Y.

